# Tetrathionate hydrolase from the acidophilic microorganisms

**DOI:** 10.3389/fmicb.2024.1338669

**Published:** 2024-01-29

**Authors:** Tadayoshi Kanao

**Affiliations:** Department of Agricultural and Biological Chemistry, Graduate School of Environment, Life, Natural Science, and Technology, Okayama University, Okayama, Japan

**Keywords:** tetrathionate hydrolase, reduced inorganic sulfur compounds, dissimilatory sulfur metabolism, S_4_-intermediate pathway, acidophiles, chemoautotroph

## Abstract

Tetrathionate hydrolase (TTH) is a unique enzyme found in acidophilic sulfur-oxidizing microorganisms, such as bacteria and archaea. This enzyme catalyzes the hydrolysis of tetrathionate to thiosulfate, elemental sulfur, and sulfate. It is also involved in dissimilatory sulfur oxidation metabolism, the S_4_-intermediate pathway. TTHs have been purified and characterized from acidophilic autotrophic sulfur-oxidizing microorganisms. All purified TTHs show an optimum pH in the acidic range, suggesting that they are localized in the periplasmic space or outer membrane. In particular, the gene encoding TTH from *Acidithiobacillus ferrooxidans* (*Af-tth*) was identified and recombinantly expressed in *Escherichia coli* cells. TTH activity could be recovered from the recombinant inclusion bodies by acid refolding treatment for crystallization. The mechanism of tetrathionate hydrolysis was then elucidated by X-ray crystal structure analysis. *Af-tth* is highly expressed in tetrathionate-grown cells but not in iron-grown cells. These unique structural properties, reaction mechanisms, gene expression, and regulatory mechanisms are discussed in this review.

## 1 Introduction

Tetrathionate (S_4_O_6_^2−^) is a type of polythionate (S*_n_*O_6_^2−^: *n* = 3–6) produced by the reaction of hydrogen sulfide with sulfite under acidic conditions, such as sulfur hot springs, acidic soil, and acid mine drainage (Karchmer, 1970; Williamson and Rimstidt, 1993). This compound is also produced by the oxidation of pyrite and other sulfide ores during abiotic and biological processes. Tetrathionate is soluble in water, in contrast to elemental sulfur, and is relatively more stable than thiosulfate at low pH, making it a common reduced inorganic sulfur compound (RISCs) in acidic environments. Therefore, tetrathionate is a suitable growth substrate for acidophilic sulfur-oxidizing microorganisms. Tetrathionate oxidation was first reported in the acidophilic iron- and sulfur-oxidizing bacterium *Acidithiobacillus ferrooxidans* (formerly *Thiobacillus ferrooxidans*) in the 1970s (Eccleston and Kelly, 1978).

**Figure 1:**
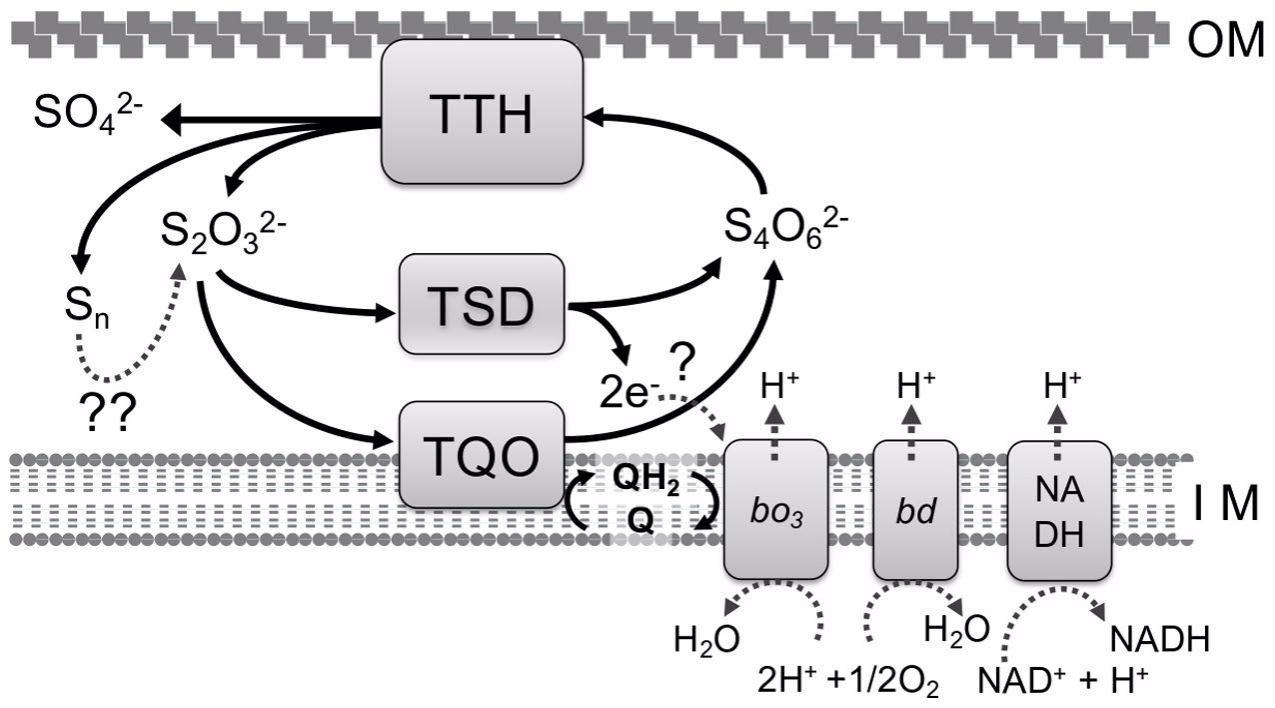
S_4_I pathway for tetrathionate and thiosulfate oxidation in *A. ferrooxidans*. TTH, tetrathionate hydrolase; TSD, thiosulfate dehydrogenase; TQO, thiosulfate:quinone oxidoreductase; Q, QH_2_, quinone pool; *bd*, *bo*_3_, terminal oxidases; NADH, NADH dehydrogenase complex I; OM, outer membrane; IM, inner membrane;?, electron acceptor;??, enzymatic and/or abiotic reactions.

Investigation of bacterial sulfur oxidation has found two major pathways for RISCs oxidation. In both pathways, thiosulfate is an important intermediate and a growth substrate for sulfur-oxidizing bacteria. The Sox system, one of the best-characterized pathways, has been investigated in the neutrophilic sulfur bacterium *Paracoccus pantotrophus* (Friedrich et al., 2001). Sox systems and their related genes are widespread among sulfur-oxidizing bacteria, including both photo- and chemoautotrophs (Hensen et al., 2006; Sato et al., 2012). The complete Sox system in *P. pantotrophus* contains four components: SoxXA, SoxYZ, SoxB, and Sox(CD)_2_. SoxXA catalyzes the oxidation of thiosulfate to generate a SoxY-cysteine-sulfhydryl group in the SoxYZ complex. SoxY acts as a thiosulfate carrier. SoxB hydrolyses the terminal sulphonate of the SoxYZ complex. Sox(CD)_2_ oxidizes the terminal sulfane sulfur of SoxY-cysteine persulfide into cysteine-S-sulfate. This system has been previously reviewed and summarized (Friedrich et al., 2005; Ghosh and Dam, 2009). Alternatively, the thiosulfate oxidation pathway (the S_4_I pathway) involves tetrathionate formation as an intermediate and is mainly observed in acidophilic sulfur-oxidizing bacteria and archaea (Figure 1). Whole-genome analysis indicates that only the S_4_I pathway is detected in the acidophilic sulfur-oxidizing bacterium *A. ferrooxidans* (Quatrini et al., 2009), even though both the Sox system and S_4_I pathway are detected in the genomes of other *Acidithiobacillus* spp., such as *A. caldus* (Mangold et al., 2011) and *A. thiooxidans* (Yin et al., 2014). This suggests that *A. ferrooxidans* mainly utilizes the S_4_I pathway for dissimilatory sulfur oxidation. In this pathway, two thiosulfate molecules are oxidized to tetrathionate by thiosulfate: quinone oxidoreductase (TQO) (Müller et al., 2004) or thiosulfate dehydrogenase (TSD) (Kikumoto et al., 2013). The membrane-bound TQO or periplasmic TSD take electrons from the oxidation of thiosulfate to the electron transport chain to produce energy or reducing power for CO_2_ fixation. Tetrathionate, a product of thiosulfate oxidation, is further metabolized by tetrathionate hydrolase (TTH). This enzyme has a unique primary structure (Buonfiglio et al., 1999) and its activity has only been detected in sulfur-oxidizing microorganisms using the S_4_I pathway for dissimilatory sulfur oxidation. TTH plays an important role in tetrathionate metabolism, not only in the S_4_I pathway, but also in the utilization of tetrathionate as a growth substrate. Here, the unique properties of TTHs are discussed to understand microbial sulfur metabolism and sulfur chemistry.

## 2 Enzyme purification and identification of TTH genes

It is well established that the acidophilic bacteria genus *Thiobacillus* can oxidize tetrathionate as an energy source. TTH was first purified and characterized in 1996 as a tetrathionate-decomposing enzyme from *A. ferrooxidans* Funis 2–1 (Sugio et al., 1996) and *A. thiooxidans* ON107 (Tano et al., 1996) (formerly *Thiobacillus ferrooxidans* and *Thiobacillus thiooxidans*, respectively). Although TTHs have been purified from *Acidiphilium acidophilum* (de Jong et al., 1997a) (formerly *Thiobacillus acidophilus*), *A. ferrooxidans* ATCC19859 (de Jong et al., 1997b), and *A. caldus* (Bugaytsova and Lindström, 2004), no genetic information on TTHs from sulfur-oxidizing bacteria was reported at that time. All TTHs described above show the maximum activity at a pH range of 2.5–4.0, which suggests that they are localized in the periplasm or on the outer membrane; different diffraction assays identified TTH from *A. caldus* as a periplasmic protein (Bugaytsova and Lindström, 2004). The properties of the TTHs from various species are summarized in Table 1. Kanao et al. (2007) purified TTH from the *A. ferrooxidans* type strain ATCC23270 and explored the N-terminal amino acid sequence of the enzyme using a BLAST search, finding that AFE_0029 encodes the N-terminal amino acid sequence. Although heterologous expression of the gene in *Escherichia coli* resulted in the formation of inclusion bodies in an inactive form, polyclonal antibodies against the recombinant protein clearly recognized the purified native TTH, indicating that the gene was immunologically identified as encoding TTH. Thus, AFE_0029 was named *Af-tth* (Kanao et al., 2007). The TTH gene from *A. ferrooxidans* is called *tetH* in several papers, however, *tet* is widely used for tetracycline-resistance genes, such as *tetK, tetL, tetM,* and *tetO* (Zilhao et al., 1988); to avoid confusion, the TTH gene will be termed *Af-tth* here. Interestingly, *Af-tth* has already been registered as a sulfur-regulated outer membrane protein from *A. ferrooxidans* MSR in the database but the function and physiological role of the gene product has not been elucidated (Buonfiglio et al., 1999). As the deduced amino acid sequence does not show similarity to any other protein in the database, this gene is reported as a novel gene encoding a sulfur-regulated outer membrane protein (Buonfiglio et al., 1999). Although *Af*-Tth is localized on the outer membrane (peripheral protein), it is detected in the culture medium supernatant, suggesting that *Af-*Tth acts in the extracellular space to generate hydrophilic sulfur globules (Beard et al., 2011). TTH genes from *A. caldus* (*Ac-tetH*) (Rzhepishevska et al., 2007) and the thermoacidophilic archaeon *Acidianus ambivalens* (*Ad-tth1*) (Protze et al., 2011) have been experimentally identified based on the amino acid sequences of purified TTHs and whole genome sequencing. The amino acid sequences of *Af-tth*, *Ac-tetH*, and *Ad-tth1* are similar and encode proteins of the pyrroloquinoline quinone (PQQ)-containing protein family (Pfam family (El-Gebali et al., 2019): PQQ_2). Inactive recombinant *Af-*Tth can be obtained in inclusion bodies from *E. coli* host cells and successfully refolded in an active form under acidic conditions without PQQ or other cofactors (Kanao et al., 2010). This provides direct evidence that *Af-tth* encodes TTH in *A. ferrooxidans* and that the enzyme does not require any cofactors for the reaction. In contrast to *Af*-Tth, quinoid compounds have been detected in *Ac*-TetH (Rzhepishevska et al., 2007). Comparison of *Ad*-TTH1 with the quinoprotein alcohol dehydrogenase from *Pseudomonas putida* showed that the heme *c* domain and conserved PQQ-binding site residues are missing, including disulfide-forming cysteines and calcium-coordinating amino acids (Protze et al., 2011). Both *Ad*-TTH1 and *Af*-Tth do not require PQQ cofactor for the reaction. Although *Ad*-TTH1 activity is detected in the pseudo-periplasmic fraction, TTH is secreted extracellularly to form zipper-like particles in the closely related thermoacidophilic archaeon *Acidianus hospitalis* (Krupovic et al., 2012). Kanao et al. (2018) identified the TTH gene from the marine acidophilic *Acidithiobacillus* sp. strain SH (SH-*tth*) using partial purification and internal peptide sequences of the protein. Although recombinant SH-Tth can be synthesized in inclusion bodies during *E. coli* heterologous expression, TTH activity is successfully recovered by acidic refolding. Interestingly, SH-Tth is activated in the presence of 1 M NaCl. This indicates that the enzyme is from a marine bacterium.

**Table 1:** Properties of TTHs isolated from various microorganisms.

Species	Molecular mass of subunit (kDa)	Optimum pH	Optimum temperature (°C)	Localization	Reference
*A. ferrooxidans* ATCC 23270	50 × 2	3.0*^1^	60*^2^	membrane	Kanao et al. (2007)
*A. ferrooxidans* ATCC 19859	52 × 2	4.0	56	periplasm	de Jong et al. (1997b)
*A. ferrooxidans* Funis 2–1	50 × 1	3.5	50	membrane	Sugio et al. (1996)
*A. caldus* KU (ATCC 51756)	52 × 2	3.0	40	periplasm	Bugaytsova and Lindstrom (2004)
*A. thiooxidans* ON 107	58 × 2	3.0–3.5	40	periplasm	Tano et al. (1996)
*Acidithiobacillus* sp. SH	52 × 2	3.0*^3^	55*^3^	membrane	Kanao et al. (2018)
*Ap. acidophilus* DSM 700	48 × 2	2.5	65	periplasm	de Jong et al. (1997a)
*Ad. ambivalens* DSM 3772	54 × 2*^4^	1.0	95*^5^	pseudo-periplasm	Protze et al. (2011)

*^1^pH stability; more than 90% activity remained even after exposure to a strong acidic buffer (0.1 M Glycine-HCl buffer pH 1.0) for 1 h on ice.

*^2^Heat stability; the enzyme displayed a half-life of 75 min at 60°C in presence of 400 mM ammonium sulfate.

*^3^Activity was assayed in the presence of 0.4 M NaCl.

*^4^Activity was detected in both the homodimer and monomer fractions.

*^5^Activity can be determined up to 95°C.

## 3 Expression, regulation, and deficiency of TTH genes

*Af-tth* expression differs depending on the growth substrate used. Quantitative RT-PCR analysis of total RNA from *A. ferrooxidans* cells grown on ferrous iron (Fe^2+^), elemental sulfur (S^0^), and tetrathionate (S_4_) showed the *Af-tth* expression ratios of S^0^/Fe^2+^ and S_4_/Fe^2+^ are 68 ± 21 and 181 ± 5, respectively (Kanao et al., 2023). This result suggests that gene expression is upregulated in cells grown on elemental sulfur and tetrathionate, but not on ferrous iron. The *Ac-tetH* expression ratio of S_4_/S^0^ is 233.5 ± 134.0 (Rzhepishevska et al., 2007). *Ac-tetH* from *A. caldus* is also highly expressed in cells grown on tetrathionate compared to cells grown on elemental sulfur. TTH activity is detected in tetrathionate-grown cells, but not in elemental sulfur-grown cells of *Ad. ambivalens*. Northern blot hybridization analysis supports the enzyme activity results, which show a much stronger signal of *Ad-tth1* in tetrathionate-grown cells and a faint signal in elemental sulfur-grown cells. This suggests that *Ad-tth1* transcription is also upregulated depending on the growth substrate, similar to *Ac-tetH* expression in *A. caldus*.

The *Ac-tetH* gene cluster contains two component system-related genes (*rsrS* and *rsrR*) and a TQO-coding gene (*doxD*) (Rzhepishevska et al., 2007). Gene expression is regulated by a two-component system called the RsrS-RsrR system (Wang et al., 2016). The relative transcriptional levels of *Ac-tetH* in *rsrR* or *rsrS* knockout mutants are much lower than those in the wild-type when stimulated with potassium tetrathionate, indicating a positive effect of RsrS-RsrR on the transcriptional regulation of *Ac-tetH*. However, no *rsrS* and *rerR* disruption effects are observed during elemental sulfur growth. Furthermore, *Ac-tetH* deficient mutant strains cannot grow on tetrathionate (van Zyl et al., 2008). These results suggest that *Ac*-TetH is the sole enzyme that metabolizes tetrathionate in the S_4_I pathway and that the Sox system may function in the elemental sulfur-grown cells of this bacterium.

A stoichiometric model of RISCs oxidation has been proposed for *A. thiooxidans* (Bobadilla Fazzini et al., 2013). According to this model, although tetrathionate is generated by the TQO (DoxDA) reaction from thiosulfate, it is chemically reduced to elemental sulfur and thiosulfate in sulfur-grown cells. Thus, no metabolic flux by way of TTH is expected, and the Sox system mainly functions during elemental sulfur growth. A similar flux is observed, assuming the presence of elemental sulfur in the TTH reaction, even in cells grown on tetrathionate. When TTH without sulfur formation functions in tetrathionate-grown cells, sulfur oxidation occurs mainly via the S_4_I pathway, and TTH plays a central role in *A. thiooxidans*.

Two-component transcriptional regulation systems (RegB/RegA) have been previously investigated in *A. ferrooxidans* (Moinier et al., 2017). According to this study, the −12/24 element and predicted integration host factor (IHF) binding sites are also proposed. IHF is expected to bind to the region upstream of the −12/−24 element and regulate gene expression with a sigma-54 dependent transcriptional response regulator (AFE_0027). Yu et al. (2014) constructed *Af-tth* knockout and overexpression strains and showed that the *Af-tth* knockout strain could not grow on tetrathionate medium, suggesting that *Af-*Tth is the sole enzyme responsible for tetrathionate hydrolysis. However, cell growth of the knockout mutant was observed on elemental sulfur with a slightly lower cell yield than that of the wild-type. Genes related to Sox systems could not be assigned to the whole-genome sequence of *A. ferrooxidans*. Some sulfur oxidation systems unrelated to the S_4_I pathway or Sox system should exist in this bacterium. In contrast, the highest and lowest growth yields were observed in the *Af-tth* overexpression strain on tetrathionate and elemental sulfur, respectively. Thus, *Af*-Tth influences sulfur metabolism to some extent in *A. ferrooxidans*.

## 4 Expression vectors utilizing *tth* promoters

Zhang et al. (2014) identified the *Ac-tetH* promoter (P*_tetH_*) and constructed an expression vector utilizing P*_tetH_* and containing the eGFP gene to confirm the functionality of P*_tetH_*. Although recombinant *E. coli* harboring the expression vector emitted fluorescence regardless of the presence of tetrathionate, eGFP fluorescence was observed following tetrathionate induction in recombinant *A. caldus*. These results suggest that P*_tetH_* is constitutively active in *E. coli* host cells and is inducible in native host cells. For the *Af-tth* promoter (P*_tth_*), Kanao et al. (2023) constructed the expression vector pMPJC with exogenous *gfp* under P*_tth_* control using *A. ferrooxidans* host cells. Green fluorescence was observed in recombinant *A. ferrooxidans* grown on tetrathionate, but not in recombinant *E. coli* distinct from the P*_tetH_* expression vector. This suggests that P*_tth_* does not function in *E. coli* and is suitable for the *in vivo* amplification of its expression vector, even if it contains genes encoding proteins that are toxic to *E. coli* host cells.

Heterologous recombinant gene expression using *E. coli* is a valid and profitable strategy to investigate the gene products of *A. ferrooxidans*; functional recombinant gene products can provide direct evidence of gene function. However, owing to differential growth conditions, particularly pH, it is frequently impossible to obtain a recombinant protein in its functional form. Recombinant *Af*-Tth is synthesized in inclusion bodies in an inactive form in *E. coli* cells. Kanao et al. (2023) examined homologous expression of *Af-tth* modifying His-tag codon using the endogenous promoter P*_tth_* in *A. ferrooxidans*. The His-tag-modified *Af*-Tth was successfully obtained from native cells in its active form. The recombinant enzyme was easily purified by metal cation affinity column chromatography and the specific activity (2.2 ± 0.37 U·mg^−1^) was equivalent to the refolded *Af*-Tth (2.5 ± 0.18 U·mg^−1^).

## 5 Catalytic reaction of TTHs

A catalytic reaction for TTH has been proposed in experiments with intact *A. ferrooxidans* cells (Steudel et al., 1987; Hazeu et al., 1988). The first suggested reaction is:


$$S_{4}{O_{6}}^{2-}+H_{2}O\to HS_{2}S{O_{3}}^{-}+HS{O_{4}}^{-}\;\mathrm{Reaction}\;\left(1\right)$$

This tetrathionate hydrolysis reaction has been proposed for an enzyme purified from *A. ferrooxidans* (Beard et al., 2011). One molecule of tetrathionate is hydrolyzed by *Af*-Tth to generate disulfane monosulfonic acid (DSMSA) and sulfuric acid (sulfate). DSMSA is a key intermediate in the biological oxidation of pyrite (Schippers et al., 1996). As DSMSA is highly reactive, several abiotic reactions may occur spontaneously. For example, four DSMSA molecules can elongate to form elemental sulfur (S_8_) and sulfurous acid (sulfite). Consequently, polythionates, S_8_, and sulfites are the final products (Pronk et al., 1990; Wentzien et al., 1994). The second reaction (Meulenberg et al., 1992) suggests that one tetrathionate molecule is hydrolyzed to thiosulfate, sulfur, and sulfate in equimolar amounts by TTH according to the following equation:


$$S_{4}{O_{6}}^{2-}+H_{2}O\to S_{2}{O_{3}}^{2-}+S^{0}+S{O_{4}}^{2-}+2H^{+}\;\mathrm{Reaction}\left(2\right)$$

Reaction estimations using purified enzymes of *A. thiooxidans* (Tano et al., 1996) and the *A. ferrooxidans* strain Funis 2–1 (Sugio et al., 1996) are:


$$4S_{4}{O_{6}}^{2-}+5H_{2}O\to 7S_{2}{O_{3}}^{2-}+2S{O_{4}}^{2-}+10H^{+}\;\mathrm{Reaction}\left(3\right)$$

However, this equation is based on the ratio of tetrathionate consumption to thiosulfate production. Although the reaction mixture is turbid owing to the generation of elemental sulfur globules, elemental sulfur does not take into account the TTH enzymatic reaction product but was considered a secondary abiotic reaction product. HPLC analysis of *Ac*-TetH reaction products by Bugaytsova and Lindström (2004) demonstrated the presence of thiosulfate and pentathionate, suggesting the reaction:


$$2S_{4}{O_{6}}^{2-}+H_{2}O\to S_{2}{O_{3}}^{2-}+S_{5}{O_{6}}^{2-}+S{O_{4}}^{2-}+2H^{+}\;\mathrm{Reaction}\left(4\right)$$

Concerning the *Af*-Tth reaction, Kanao et al. (2007) detected the turbidity of the reaction mixture by elemental sulfur production, and the same level of thiosulfate production accompanying tetrathionate consumption was detected using the ion-pair HPLC method. Except for thiosulfate, no other polythionate peaks, such as tri-, penta-, or hexathionate peaks, were detected in the reaction mixture after the complete consumption of tetrathionate. This result suggests that the overall *Af*-Tth reaction is the same as Reaction (2). Because sulfur atoms (S^0^) are unstable, Reaction (2) should be repeated eight times to produce S_8_ as a stable end product, according to the following equation:


$$8S_{4}{O_{6}}^{2-}+8H_{2}O\to 8S_{2}{O_{3}}^{2-}+S_{8}+8S{O_{4}}^{2-}+16H^{+}\;\mathrm{Reaction}\;\left(5\right)$$

Determining the specific reaction of the enzyme is difficult because tetrathionate hydrolysis in *Acidithiobacillus* generates multiple products with chemical interactions between thiosulfate, sulfane sulfur chemicals, polythionate, sulfate, and possibly S_8_ at high sulfate concentrations and low pH. In addition, *Af*-Tth exhibits a unique primary structure, and the catalytic reaction is unprecedented. Therefore, the precise whole reaction and reaction mechanism of TTH remain unknown.

Cysteine residues in the catalytic center play important roles in many other enzymes involved in dissimilatory sulfur oxidation, including sulfide:quinone oxidoreductase (Cherney et al., 2010), sulfur oxygenase reductase (Veith et al., 2011), and thiosulfate dehydrogenase (Brito et al., 2015). Although cysteine residues are normally conserved in each enzyme among different sulfur-oxidizing microorganisms, only one cysteine residue (Cys301) can be detected in the primary structure of *Af*-Tth and is not conserved among TTHs from *Acidithiobacillus* spp. (Kanao et al., 2014). Moreover, the site-specific variant (*Af*-Tth C301A) exhibits similar activity and dimeric formation. These results suggest that the sole cysteine residue (Cys301) in *Af*-Tth is not involved in the tetrathionate hydrolysis reaction or subunit assembly, indicating a novel cysteine-independent reaction mechanism for this enzyme.

## 6 Reaction mechanism of tetrathionate hydrolysis

**Figure 2:**
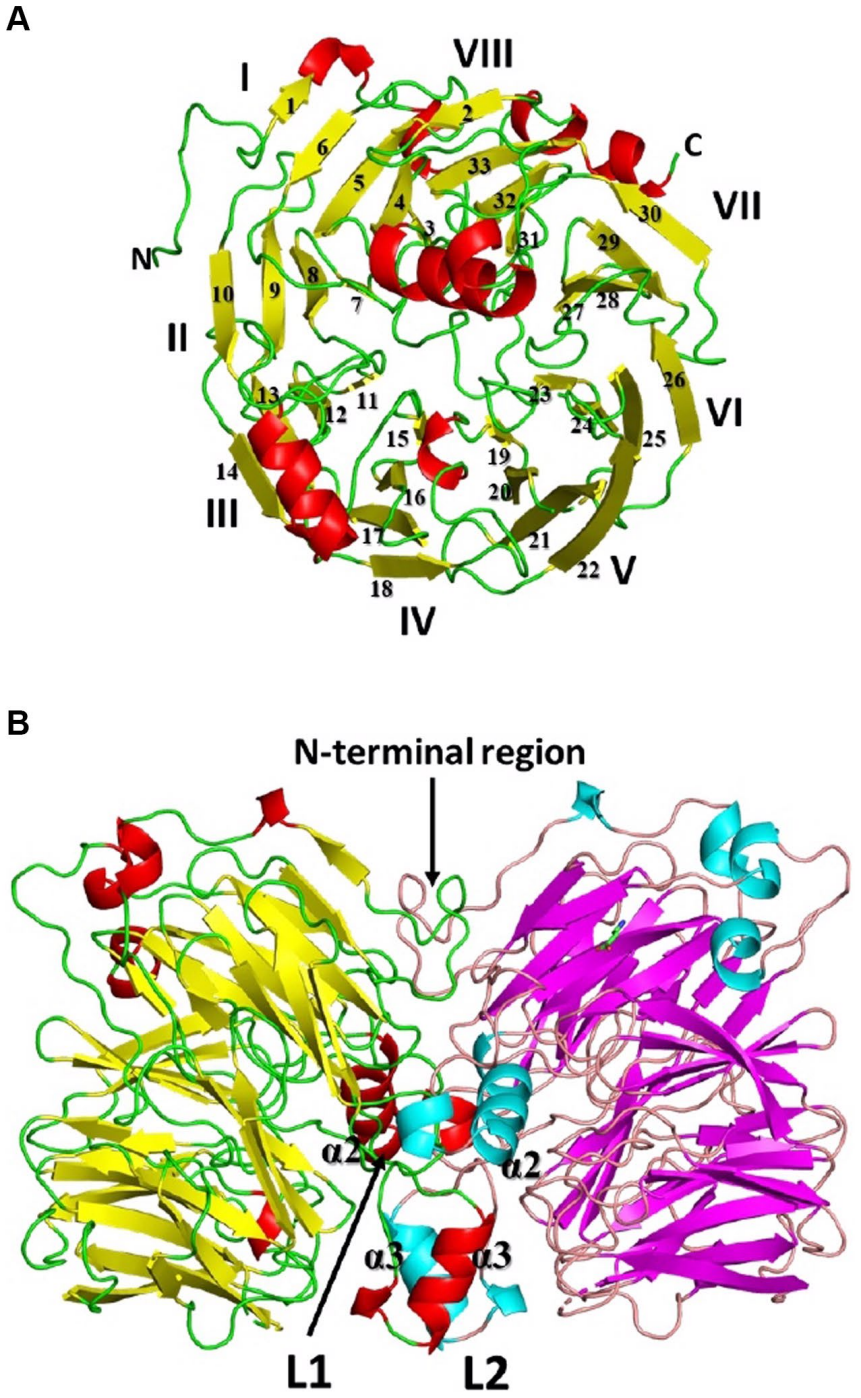
Overall structure of recombinant *Af*-Tth. **(A)** Monomer and **(B)** dimer structures. For each monomer of chains B and D in the dimer, beta-strands are colored yellow and magenta, alpha- and 3_10_ helices are red and cyan, and loops are green and wheat, respectively. The monomer in **(A)** is the view from the right side of **(B)**. The monomer structure exhibits an eight-bladed (I-VIII) beta-propeller motif. L1 and L2 are the insertion loops of the eight-bladed beta-propeller motif.

**Figure 3:**
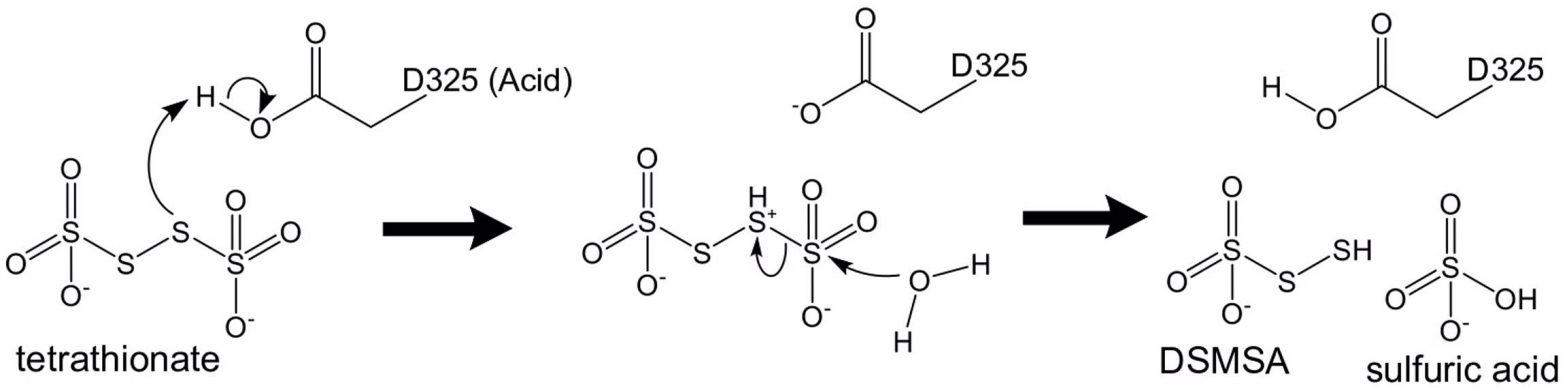
Proposed catalytic mechanism of tetrathionate hydrolysis by *Af*-Tth. The amino acid residue, Asp325, works as a general acid under neutral or acidic conditions. DSMSA, disulfane monosulfonic acid.

Recombinant *Af*-Tth crystals (hexagonal cylinder with dimensions of 0.2 mm × 0.05 mm × 0.05 mm) have been successfully obtained (Kanao et al., 2013) and the X-ray crystallographic results reported (Kanao et al., 2021). *Af*-Tth is a homodimer and its monomer structure exhibits an eight-bladed beta-propeller motif, as determined by X-ray crystallography at 1.95 Å resolution. Two insertion loops participate in dimerization, and one loop forms a cavity with the beta-propeller region (Figure 2). Analysis of the substrate-soaked structure of *Af*-Tth has shown the electron density of the polymerized sulfur atoms derived from the tetrathionate molecules and the catalytic center of the enzyme. The sole cysteine residue (Cys301) is located >25 Å from the active site. Asp325 of *Af*-Tth located within 4 Å of the electron density is conserved among TTHs from *Acdithiobacillus* spp. and *Ad*-TTH1 from *Ad. ambivalens*. TTH activity is completely abolished in a site-specific *Af*-Tth D325N variant, suggesting that Asp325 plays a crucial role in the first tetrathionate hydrolysis step. Considering that the *Af*-Tth reaction occurs only under acidic pH conditions, Asp325 acts as an acid for the tetrathionate hydrolysis reaction (Figure 3). If Asp325 acted as an acid, the S_α_ atom of the tetrathionate molecule could be protonated by Asp325. Owing to the protonated S_α_ atom, the terminal sulfur atom of the tetrathionate molecule is easily hit by the nucleophilic attack of a water molecule. The sulfur–sulfur bond between the terminal sulfur atom and protonated S_α_ atoms is broken in an S_N_2 reaction, thereby generating sulfate and DSMSA. This is consistent with the Reaction (1). The next step in the *Af*-Tth catalytic reaction is proton abstraction from DSMSA, followed by the release of a sulfur (S^0^) atom:


$$HS_{2}S{O_{3}}^{-}\to S_{2}{O_{3}}^{2-}+S^{0}+H^{+}\;\mathrm{Reaction}\;\left(A\right)$$

Reaction (2) is obtained by combining Reaction (1) and (A). Moreover, the *Af*-Tth reaction is multistep. The released sulfur (S^0^) atoms oligomerize and finally cyclize as S_8_. However, the underlying mechanisms remain unknown. Further studies are required to elucidate the complete reaction mechanism of this unique enzyme.

## 7 Discussion

To date, various enzymes related to RISC oxidation metabolism have been investigated, but most are oxidoreductases, such as SoxXA, Sox(CD)_2_, TQO, TSD, sulfide:quinone oxidoreductase, sulfur dioxygenase, and sulfur oxygenase reductase, and a few hydrolases (only TTH and SoxB (Sauvé et al., 2009)). TTH, which catalyzes tetrathionate hydrolysis, is a unique enzyme that cooperates with TQO and/or TSD to function in the S_4_I pathway for RISC oxidation, particularly of thiosulfate (Figure 1). The S_4_I pathway is widely distributed in alpha-, beta-, and gamma-proteobacteria, in particular acidophilic and neutrophilic sulfur-oxidizing bacteria, including the genera *Acidiphilium*, *Tetrathiobacter*, *Acidithiobacillus*, and *Thermithiobacillus* and thermoacidophilic archaea belonging to *Sulfolobaceae* including the genus *Acidianus*. However, TTHs have only been purified and characterized from acidophilic autotrophic sulfur-oxidizing microorganisms (Wang et al., 2018). All purified TTHs show an optimum pH in the acidic range, suggesting that they are localized in the periplasmic space or outer membrane (Table 1). TTH catalyzes a unique cysteine-independent reaction. Elucidating the detailed reaction mechanism of how the enzyme cleaves the sulfur–sulfur bond in the DSMSA molecule is of interest from the viewpoint of both inorganic sulfur chemistry and enzymology. It is expected that detailed X-ray crystallographic analyses of both site-specific variants and wild-type enzymes will clarify the complete reaction mechanism of TTH.

TTH gene expression is regulated and enhanced by a two-component system in cells grown on tetrathionate in *Acidithiobacillus* spp. and *Ad-tth1* expression is also regulated at the transcriptional level depending on the growth substrate. Although no homologous genes related to the Sox system were detected in the whole-genome sequence of *A. ferrooxidans*, *Af-tth* knockout variants showed good growth on elemental sulfur. This suggests that other RISC oxidation pathways may be distinct from the Sox and S_4_I pathways. For instance, TTH independent tetrathionate metabolism for thiosulfate oxidation was recently reported in the newly isolated bacterium, *Erythrobacter flavus* (Zhang et al., 2020). In addition, although the strain closely related to *Acidicaldus* sp. DX-1 can grow on tetrathionate overlay medium (Johnson et al., 2006), no candidate genes with significant similarity to *tth* and Sox relevant genes were found in the draft genome of the strain DX-1 (Liu et al., 2006). Investigation of microbial sulfur oxidation, especially in acidophilic sulfur-oxidizer, is essential and important not only for their applications but also for understanding the sulfur cycle in the environment.

## Author contributions

TK: Writing – original draft, Writing – review & editing.
